# Beyond the Brain Song: Can Music Help Us Teach Undergraduate Neuroscience? A Review of *From Perception to Pleasure: The Neuroscience of Music and Why We Love It*

**DOI:** 10.59390/001c.154550

**Published:** 2025-12-31

**Authors:** Lincoln G. Craton, John G. McCoy

**Affiliations:** 1 Department of Psychology Stonehill College

**Keywords:** music, book review, perception

The neuroscience of music is advancing quickly. A simple “music and brain” search in PubMed reveals an exponential increase in published articles in the past three decades, from 263 (1990s) to 1,183 (2000s) to 2,628 (2010s). Edited tomes such as *The Oxford Handbook of Music and the Brain* (Hodges & Thaut, 2021) provide a portal into this broad literature, expertly distilling the evidence but necessarily glossing over the methodological details. More engaging and accessible for undergraduate readers are *This is Your Brain on Music* (2006) and Oliver Sacks’ *Musicophilia* (2007), delightful old standbys that unfortunately predate important new work on the auditory circuitry and reward processes underlying musical experience.

Into the mix comes *From Perception to Pleasure: The Neuroscience of Music and Why We Love It*[Bibr ref-530145], a timely magnum opus that readers of this journal may find to be an exciting new resource. Robert Zatorre, an influential neuroscientist whose passion for research sings from every page, has given us an engrossing and lavishly illustrated account of the neural basis of music and its enjoyment. *From Perception to Pleasure* is, as Zatorre describes in the preface, an academic book that is “reasonably readable by interested, nonspecialist readers, but without sacrificing detailed content…there’s a bit of a scientific memoir embedded in there along with all the graphs and brains.” This hybrid approach makes for a challenging yet inviting read. Zatorre has a cutting-edge scientific story to tell, and he does so lucidly, with lots of engaging examples and a bit of narrative drama. The many gorgeous colorful illustrations of “graphs and brains” also add immensely to the book’s appeal.

Despite its rising popularity, music neuroscience remains for most of us a bit of a niche topic and feels like a low priority for the classroom. In years past, one of us (LC) enjoyed juxtaposing music and brain in class using a rousing comic interlude called *The Brain Song*. But that was about it (sadly, a current online search for “brain song” yields truly unlistenable performances or eyebrow-raising links to a self-help product). Zatorre’s book suggests that now is the time for that to change. The following overview is intended to encourage and excite instructors who might view this as a creative opportunity.

Zatorre’s pedigree is certainly not in question. With over 300 scientific papers on pitch perception, musical imagery, music production, brain plasticity, hemispheric specialization, and the role of the reward system in musical pleasure, he has contributed generously to the empirical growth of music neuroscience over the past 40 years. The book tells the story of this work, conducted at McGill University and the Montreal Neuro Institute (MNI), a historically and currently important place for neuroscience that continues to produce some of the most groundbreaking work on music neuroscience in the world. This is where Wilder Penfield, Donald Hebb, Brenda Milner (alive and kicking at 106), James Olds, and Peter Milner made their discoveries.

*From Perception to Pleasure* is divided into two parts—on perception and pleasure, of course—reflecting Zatorre’s overarching thesis that “musical pleasure…arises from the functional interactions between two distributed and complex neural systems: the perceptual/motor/cognitive system on the one hand and the reward system on the other” (p. 4). After an opening chapter describing early auditory processes occurring in subcortical, core auditory, belt and parabelt areas, Part I devotes independent chapters to two major pathways: the ventral and dorsal streams. Both streams consist of “corticocortical loops” in each cerebral hemisphere. But whereas the ventral stream is specialized for working memory retention, generating internal representations of musical structure, and detecting deviations from expected musical events, the dorsal stream is specialized for transforming information in working memory in order to perceive, compose, or produce patterns of musical sounds. A closer look at each chapter in Part I shows why the book is a promising resource for anyone wishing to cover music perception/cognition in their class.

The first chapter in Part I by itself justifies the book’s purchase price ($30). Deftly framing the computational problem our auditory system solves in order to recover the universe of sound from the jumble of sound waves impinging on the eardrum, the narrative quickly proceeds to a review of the most important early processes in brainstem nuclei and the auditory cortex: encoding of acoustic cues, adaptation, the frequency-following response, segregation of sound sources, tonotopic frequency organization, pitch representation, perception of nonmusical sounds, attentional phenomena, musical imagery, and musical hallucinations. Students may be particularly interested in Nina Kraus and colleagues’ work on the frequency-following response showing that musicians have a higher amplitude FFR that supports better pitch representation and an increased ability to perceive speech in a noisy environment.

The chapter on the auditory ventral system begins by tracing the history of lesion effects and neuroimaging data showing the role of auditory cortex and frontal regions for music processing and auditory working memory. The ensuing discussion of contemporary work is truly exciting. Like us, students will likely be amazed to learn about fMRI work by Sam Norman-Haignere and colleagues that points to a temporal cortical region that is responsive only to music. The discovery of a music-specialized module strikes us as a major advance and, along with evidence for a separate vocal-selective area, has huge implications for the origin of music. Also, Zatorre’s excellent presentation of congenital amusia—yes, there are individuals who are selectively unable to perceive music—as a disorder of the auditory ventral stream is fascinating.

The dorsal stream chapter explores a separate pathway that proceeds from auditory cortex to parietal, premotor, and other dorsal regions of the frontal lobe. This circuitry appears to supplement the straightforward representations of the ventral stream by generating more abstract and flexible representations that listeners can manipulate. Take, for instance, our natural ability to transpose a melody based on the relation of the pitches to each other, so that *Happy Birthday* can be recognized regardless of the absolute pitches of the notes. By making it possible for listeners to reorganize musical elements in pitch or in time (rhythm), the dorsal stream underlies our ability to appreciate the many, often subtle, variations that occur during a musical performance. In addition, by engaging the motor system while singing or playing a musical instrument, this pathway underlies musical performance in all its many forms. Students will be amused to learn about the invention by Zatorre and colleagues of the world’s first cello that can be played inside an MRI scanner! The ensuing research has supported a “neural recycling” account of instrument playing, in which dorsal system structures that evolved to support singing are co-opted to support the cognitive operations needed to play cello. Musicians in particular will relate to the notion that we really do sing with our instruments.

A final chapter in Part I, although less integrated with the overarching story of the book, presents a helpful account of the voluminous data on hemispheric specialization. Zatorre argues convincingly that, whereas speech perception relies largely on temporal cues that are best processed in the left hemisphere, music perception is more dependent on spectral information that is best processed in the right hemisphere.

Part II of the book explores why we love music and the neural substrates involved. While individual preferences for musical genres and styles abound, the experience of music as pleasurable is nearly ubiquitous, and certainly spans diverse cultures. Most people at least occasionally experience intensely pleasurable “chills” from music, suggesting that music may serve one or more adaptive functions that facilitate individual or species level (i.e., reproduction) survival. Similar in some ways to the phenomenon of sleep, identification and proof of the actual function(s) served by music remains an elusive goal.

Zatorre’s approach to musical pleasure is helpfully systematic. He begins with a chapter on the functional anatomy of the dopaminergic reward system outside the context of music and follows this with a chapter describing recent empirical research showing that, similar to primary rewards such as food and secondary rewards like money, the abstract and aesthetic stimulus of music activates this reward system. A third chapter then draws on predictive coding theory (PCT) to develop a model for why this is so.

PCT plays a central role throughout the book, but especially in Part II. It has become a popular way to understand perception in general and Zatorre’s application to music may be an effective way to present this material in class. Roughly, the idea is that prior listening leads to the creation of an internal model that reflects the statistical likelihood of musical events. As we listen to a piece of music, we employ this model to predict, in top-down fashion, what will happen next and when. Of course, overly predictable music can become bland (i.e., habituation); yet, a single well-placed surprising note once again activates arousal, attentional and emotional brain circuits, not unlike our response to other novel stimuli in the environment.

Because the auditory pathways and frontal/parietal circuits discussed in Part I are connected to the reward structures described in Part II, successful predictions of musical resolutions evoke pleasure. How incredible that music involves ancient neural circuitry in addition to evolutionarily newer neocortical circuits! Specifically, it activates the limbic circuitry that can facilitate anticipatory tension (i.e., wanting) followed by a satisfying resolution (i.e., liking or satiation). Zatorre reviews evidence suggesting that dopamine plays a prominent role in the former (i.e., wanting/anticipation) while opioid peptides may be involved in modulating the latter (i.e., liking/resolution). It is a fairly complicated story and no doubt the truth will be even more nuanced, but Zatorre provides us with a starting point.

PCT is not the only game in town (cf. Harding et al., 2025). But in Zatorre’s hands it does a good job explaining why we are sensitized to novel musical stimuli and how neuroplasticity allows for modification of neural circuits resulting in novel predictions while listening to musical passages. Just as novel foods can become an “acquired taste,” we use the same mesolimbic circuitry as we come to appreciate a new musical form. One need not buy into PCT to appreciate Zatorre’s mind-blowing, student-friendly descriptions of specific musical anhedonia and the search for its neural correlates. That’s right, roughly 5% of participants tested derive no enjoyment from music even though they enjoy food, touch, or sex. What’s more, this condition can be recreated in music lovers using transcranial stimulation!

In a final major chapter entitled “pleasure and beyond,” Zatorre stirs in the active area of music and emotion, and emotional regulation.

Clearly, we recommend *From Perception to Pleasure* as a personal read for researchers and other interested faculty. It illustrates the significant progress being made in developing an empirically supported neuroscience of music, and Zatorre’s particular theoretical framework provides testable hypotheses for researchers in the area as well as for informed cheerleaders like ourselves. For instance, one prominent topic not covered in the book is the role of the default mode network (DMN). The DMN is active during quiet wakefulness and deactivated during goal-directed behaviors. So many great composers have remarked that their best songs seem to require little effort, with the melody arising seemingly out of nowhere. One wonders if the DMN plays a significant role in these instances?

We think that the book could have educational utility at the undergraduate level. How would we bring it to budding young neuroscientists? Although intended for nonspecialists, it presumes some degree of familiarity with neuroanatomy and neuroscience in general. A prerequisite course in biopsychology (also known as behavioral neuroscience, physiological psychology, brain and behavior, etc.) would be recommended. With that as background, we can see it being a centerpiece for a fantastic senior seminar class. Alternatively, the juiciest material (see our examples above) might be cherry-picked and incorporated into lectures in a standard biopsychology course.

Finally, many institutions offer interdisciplinary courses and/or courses from separate disciplines organized around a central theme. These offer students the advantage of viewing a topic from separate perspectives and from disciplines that employ different research techniques, each with its own advantages and limitations. At our college, courses linked in this way are referred to as Learning Communities. Zatorre’s book is ideally suited for such a purpose. For example, a Learning Community consisting of coursework and faculty from neuroscience, psychology and music departments could benefit greatly from Zatorre’s book. A flipped-classroom format in which students complete reading assignments prior to class, with discussions ensuing in class sounds like a course we would have loved to have taken.

Zatorre’s book provides a cogent summary of findings in the burgeoning field of music neuroscience, while awakening the curiosity and imagination of all those interested in how and why it is that music plays such an integral part of our lives. Like a sophisticated piece of music, *From Perception to Pleasure* provides a challenging but increasingly gratifying experience as one works through it. Evidently, music neuroscience itself can activate the reward system!
